# Quantitative assessment of the oral microvasculature using optical coherence tomography angiography

**DOI:** 10.3389/fbioe.2024.1464562

**Published:** 2024-09-20

**Authors:** Tianyu Zhang, Yilong Zhang, Jinpeng Liao, Simon Shepherd, Zhihong Huang, Michaelina Macluskey, Chunhui Li

**Affiliations:** ^1^ Centre for Medical Engineering and Technology (CMET), School of Science and Engineering, University of Dundee, Dundee, United Kingdom; ^2^ Healthcare Engineering, School of Physics and Engineering Technology, University of York, York, United Kingdom; ^3^ School of Dentistry, University of Dundee, Dundee, United Kingdom

**Keywords:** optical coherence tomography, angiography, quantitative analysis, oral squamous cell carcinoma, OCTA, intraoral imaging, oral microcirculation

## Abstract

**Introduction:**

Early diagnosis of oral squamous cell carcinoma can greatly improve treatment success rate and patient survival. Although Optical Coherence Tomography (OCT) based Angiography (OCTA) is a promising in vivo technique in oral imaging, there is a need for objective assessment of oral microvasculature.

**Methods:**

This study aimed to demonstrate a comprehensive methodology of quantitative assessing OCTA intraoral scanning results to provide measurable, reproducible data and to avoid subjective visual interpretations. Data were collected from 37 healthy subjects in total across four intraoral sites—buccal mucosa (n = 32), labial mucosa (n = 24), floor of the mouth (n = 13), and hard palate (n = 8)—using a non-invasive swept-source OCT system. Four quantitative metrics—vessel area density, vessel skeleton density, vessel diameter index, and a newly proposed weighted Tortuosity Index—were used to assess OCTA images in oral applications.

**Results:**

The quadruple quantitative assessment’s repeatability was evaluated to be reliable. Analysis of a benign ulcer case revealed differences in these metrics compared to healthy cases.

**Discussion/Conclusion:**

In conclusion, we demonstrated a comprehensive method to quantify microvasculature in the oral cavity, showing considerable promise for early diagnosis and clinical management of oral diseases.

## 1 Introduction

Oral squamous cell carcinoma (OSCC) represents a significant health concern due to its aggressive nature and adverse outcomes (Chamoli et al., 2021). Early diagnosis is crucial in managing OSCC, as it greatly reduces morbidity and improves the chances of successful treatment and patient survival (Sciubba, 2001; Feller and Lemmer, 2012; Mortazavi et al., 2014). The unique vascular patterns associated with tumor growth provide critical insights into the malignancy’s progression and status (Macluskey et al., 2000; Sasahira and Kirita, 2018; Tirelli et al., 2018). Specifically, vessel density, diameter, and tortuosity were found related to oral diseases (Ravi et al., 1998; Djaberi et al., 2013; Sasahira and Kirita, 2018). In this context, the study of microvasculature within OSCC lesions has emerged as a promising diagnostic avenue. Imaging modalities for assessing the oral microvasculature have seen significant developments in past decade, and has included high-frequency ultrasound (Huang et al., 2017; Fogante et al., 2022), real-time optical vascular imaging (RTOVI) (Bastos et al., 2022) and video-capillaroscopy (Scardina et al., 2007). However, high-frequency ultrasound is limited by its resolution compared to optical imaging techniques, while RTOVI is challenged by a restricted field of view. Video-capillaroscopy has only a shallow penetration depth due to using visible light for imaging. These limitations may impact on the ability of these techniques to capture the nuanced vascular changes at the earliest stages of OSCC. Therefore, there is a pressing need for more advanced, non-invasive imaging technologies that can accurately visualize and quantify microvascular alterations in OSCC, facilitating early and more effective diagnosis.

Optical coherence tomography (OCT) based angiography (OCTA) is a relatively recent innovation in imaging technology which has been developed for applications in oral diagnostics (Choi and Wang, 2014; Chen and Wang, 2017; Tsai et al., 2017; Le et al., 2018; 2022; Wei et al., 2018; Zhang et al., 2023). As a non-invasive imaging technique, OCTA offers high-resolution, three-dimensional views of microvascular structures without the need for contrast agents (Kashani et al., 2017). This technology operates on the principle of capturing the motion contrast of red blood cells, thereby providing detailed images of blood flow within tissues (Chen and Wang, 2017). These emerging applications highlight OCTA’s growing significance in oral healthcare, providing a new frontier in the imaging-based assessment of oral diseases.

While OCTA, this non-invasive functional imaging technique has shown promises in oral imaging, there remains a need for objective assessment techniques of captured oral angiograms. Quantitative assessments of OCT angiograms have been implemented in other applications, e.g., in cardiology (Xie et al., 2024), dermatology (Untracht et al., 2021; Manfredini et al., 2023) and ophthalmology (Reif et al., 2012; Agemy et al., 2015; Jia et al., 2015; Chu et al., 2016; Engberg et al., 2020; Untracht et al., 2021; Wang et al., 2022), which can avoid subjective visual interpretations and provide measurable, reproducible data. For the analysis of microvascular structures, the aforementioned studies introduced several parameters, such as vessel area density (VAD) (Reif et al., 2012; Jia et al., 2015), vessel skeleton density (VSD) (Reif et al., 2012; Agemy et al., 2015), vessel diameter index (VDI) (Chu et al., 2016), and tortuosity index (TI) (Lee et al., 2018; Martelli and Giacomozzi, 2021). These metrics could bring advancements in characterizing the various vascular diseases. VAD offers insights into the density of the vascular network by measuring the area occupied by vessels (Reif et al., 2012; Jia et al., 2015; Chu et al., 2016), while VSD focuses on the length of these vessels, providing a different perspective on vascular distribution (Reif et al., 2012; Agemy et al., 2015; Chu et al., 2016). The VDI can contribute further by analyzing the average diameter of vessels (Chu et al., 2016). These parameters are vital in identifying and quantifying subtle vascular changes that may indicate disease presence or progression. However, it is important to note that each of these parameters, while valuable, might only provide a partial view of the vascular landscape. For instance, VAD and VSD might not fully capture the dynamic aspects of blood flow or the functional status of the vessels (Chu et al., 2016). Similarly, VDI, dependent on image resolution and quality, might have limitations in accurately portraying the intricate details of microvascular architecture. Despite these limitations, these metrics collectively offer a comprehensive framework for assessing and understanding vascular alterations in various pathological states using OCTA (Chu et al., 2016). In addition, the tortuosity of the blood vessels is a significant factor for physiological features in diseases, which has been studied since Leonardo Da Vinci’s works (Ciurică et al., 2019; Wells and Crowe, 2004; Kemp, 2019). A number of medical conditions or biological processes, such as aging, atherosclerosis, hypertension, genetic defects, and diabetes mellitus, can contribute to the development of increased or severe vessel tortuosity according to clinical studies (Del Corso et al., 1998; Pancera et al., 2000; Hiroki et al., 2002; Owen et al., 2008; Kahe et al., 2020). Although several metrics, TI, average TI and Vessel Complex Index (VCI), have been introduced for assessing vasculature tortuosity, none of these metrics include the factor of the vessel diameter, which is crucial for understanding the varying physiological significance of blood vessels of different diameters (Han, 2012; Yoon et al., 2023). Therefore, a novel approach to assess the vascular tortuosity factoring in the vessel diameter is necessary for quantitative assessment of microvasculature.

In our research, we have employed a set of quantitative metrics, VAD, VSD, and VDI, to assess OCTA images in oral applications. In addition, we have applied a weighted Tortuosity Index (WTI) calculation to assess the tortuosity of the blood vessels. These metrics have been chosen to provide a multi-dimensional understanding of the microvascular structures within the oral cavity to enhance the diagnostic capabilities and to offer clinicians a more nuanced view of vascular changes associated with various oral diseases. This approach enhances a detailed assessment of microvascular structures, which has the potential to contribute to improved monitoring and treatment of oral disease. In addition, to ensure that OCTA using these metrics can be effective as a diagnostic tool for diseases, it is crucial to understand the imaging within the context of healthy tissues.

## 2 Methods and materials

### 2.1 OCT system

**FIGURE 1:**
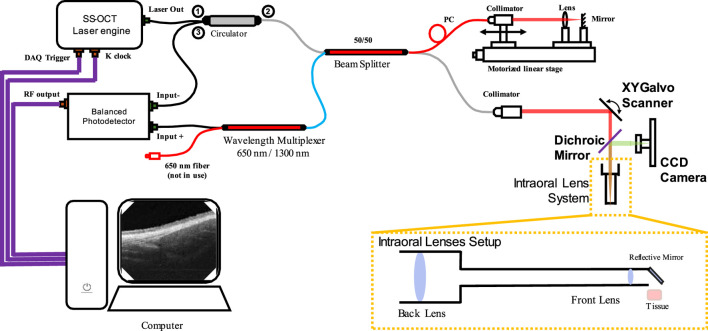
The optical diagram of the SS-OCT system used in this study. (CCD: Charge-coupled device).

The OCT system used in this study was introduced previously (Zhang et al., 2023), which was a lab-built, portable and non-invasive swept-source OCT (SS-OCT) system with a handheld scanning probe. The diagram of the SS-OCT system is shown in Figure 1. The laser source of this SS-OCT system is a vertical-cavity surface-emitting laser (VCSEL) source (SL132120, Thorlabs Inc., Newton, MA, United States), with a central wavelength of 1,300 nm, and a bandwidth of 100 nm. The imaging lens system was a two-lens system (AC127-075-C and AC254-125-C, Thorlabs Inc., Newton, MA, United States) designed for intraoral imaging, providing a lateral resolution of 39 μm. The theoretical axial resolution was 7.5 μm in air. The field of view was 5.25 mm × 5.25 mm for the intraoral scanning probe.

### 2.2 Data collection

In this study, a total of 37 healthy participants were involved in the intraoral acquisition, which included four different intraoral sites, buccal mucosa, labial mucosa, floor of the mouth and hard palate. Due to different acceptances of the intraoral acquisition among participants, each scanning location had different numbers of datasets. Specifically, the buccal mucosa acquisitions involved 32 participants. The labial mucosa acquisitions involved 24 participants. The acquisition of the floor of the mouth involved 13 participants. Lastly, the hard palate acquisitions involved eight participants. This study was reviewed and approved by the Research Ethics Committee of the University of Dundee (UOD-SSREC-RPG-BioEng-2022-001).

### 2.3 Data processing

**FIGURE 2:**
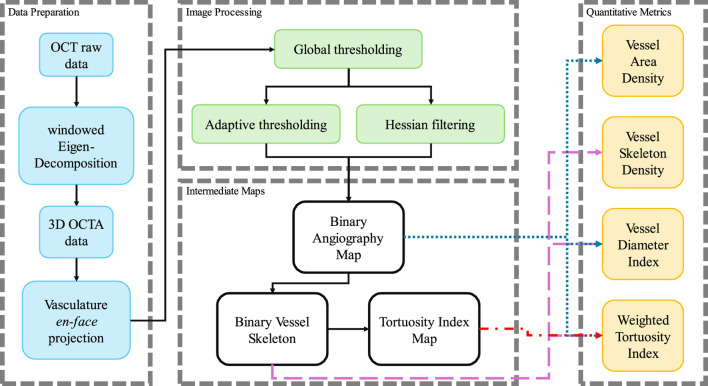
The processing flow chart in this study to process the OCTA data and calculate the quantitative metrics.

The datasets acquired by this SS-OCT system contained four dimensions (4D), including three spatial dimensions and one temporal dimension. The size of the datasets was 950 × 400 × 400 × 4 (Z × X × Y× N in pixels, where Z was the axial dimension, X and Y were the lateral dimensions, and N was the temporal dimension). Several processing steps of data preparation were required to generate the intermediate maps, which would be needed for the quantification evaluation. The preparation flowchart is shown in Figure 2.

**FIGURE 3:**
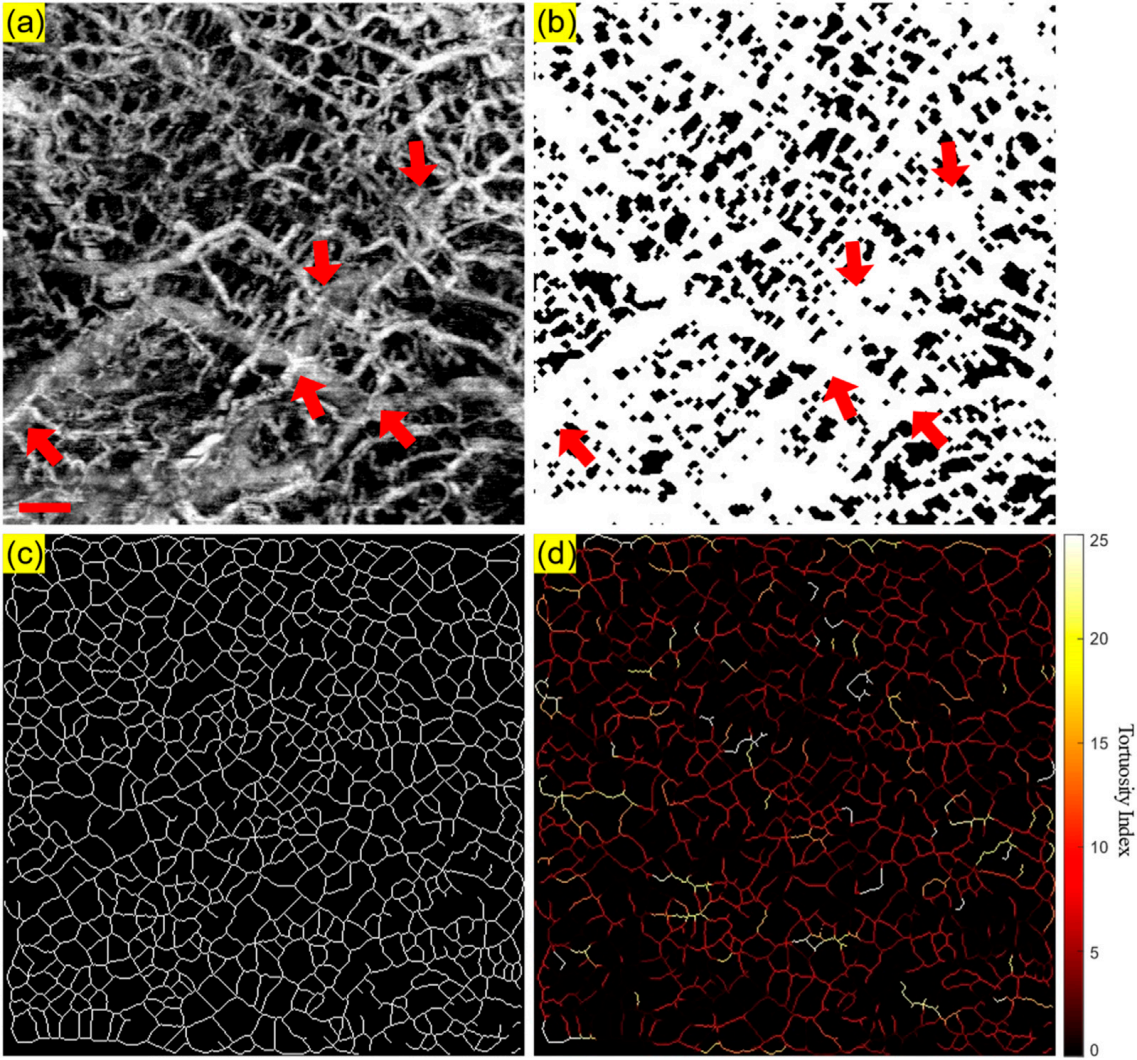
An illustrative OCTA dataset (healthy labial tissue) processed to generate the projection image and the three intermediate maps used for quantitative assessment: **(A)** The OCTA *en face* projection image with a red scale bar of 500 μm; **(B)** The binary angiography mask (BAM); **(C)** The binary vessel skeleton (BVS); **(D)** The Tortuosity Index (TI) map. The red arrows highlight the overlapping of blood vessels.

Firstly, the angiography reconstruction method, windowed Eigen-Decomposition (wED) (Zhang et al., 2022), was applied to the acquired 4D datasets, generating 3D volumes of angiography signals. Then, the 3D volumes would be compressed using Maximum Intensity Projection (Sato et al., 1998; Wang et al., 2018) to produce the *en face* projections. With the projection results of angiogram, the areas of angiography signals can be selected using global thresholding (Otsu, 1979), adaptive thresholding (Bradley and Roth, 2007), and Hessian Filter (Frangi et al., 1998; Reif et al., 2012). The Otsu’s method was used to determine the global threshold (Otsu, 1979). And then, after the combination of the adaptive thresholding (Bradley and Roth, 2007; Reif et al., 2012) and Hessian Filter (Frangi et al., 1998), the binary angiography masks (BAM) were generated and prepared for the OCTA quantification. With the BAM, the binary vessel skeletons (BVS) were generated (Lee et al., 1994; Kerschnitzki et al., 2013) while the BVS was processed to separate vessel segments and calculate the TI which generated the Tortuosity Index skeleton map. The intermediate maps are demonstrated in Figure 3. As shown in Figure 3B, BAM had the blood vessel areas as 1s, and the rest areas as 0s. BVS in Figure 3C where each blood vessel appeared as a distinct, one-pixel-wide line, contained the skeletons of the blood vessels in 1s, while the Tortuosity Index map in Figure 3D had the distribution of the TI for all vessel segments.

**FIGURE 4:**
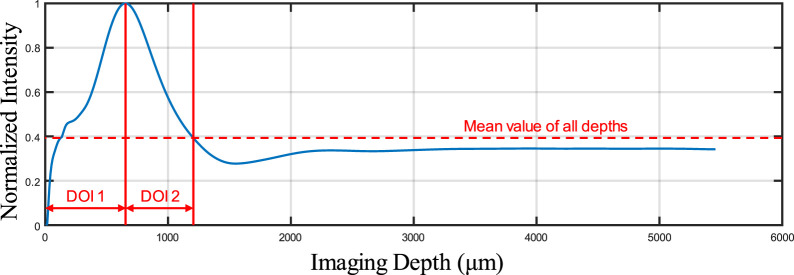
The normalized signal intensity distribution on all imaging depths.

As shown in Figure 3, some overlapping of blood vessels can be observed, which can cause inaccurate quantitative assessments, especially for BVS. Therefore, an automatic Depth of Interest (DOI) selection algorithm was developed to divide one OCTA dataset into two individual layers. As each volunteer will have different oral tissue thickness and signal attenuation, employing fixed-depth layer separation was considered an inappropriate method for solving this problem. In order to adapt to the different situations for all participants, the DOI was selected automatically depending on the average OCTA signal intensity at various depths. Specifically, for each 3D OCTA dataset of Z × X × Y in pixels, the averaging processing was applied on the *X* and *Y* axis, which can output an array of Z × 1, the intensity distribution at all imaging depths as shown in Figure 4.

**FIGURE 5:**
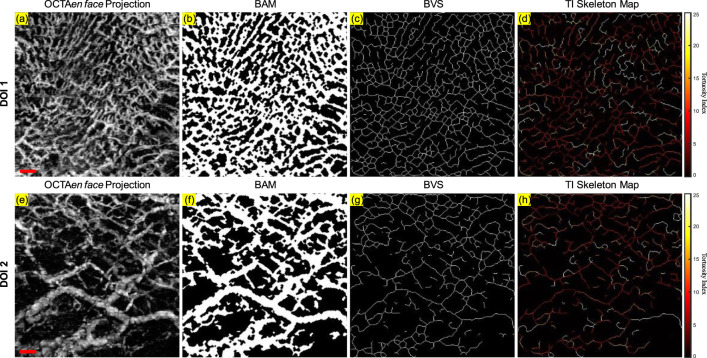
The projection image and the three intermediate maps used for quantitative assessment for both DOI 1 and DOI 2: **(A)** The OCTA *en face* projection image of DOI 1 with a red scale bar of 500 μm; **(B)** The BAM of DOI 1; **(C)** The BVS of DOI 1; **(D)** The TI skeleton map of DOI 1; **(E)** The OCTA *en face* projection image of DOI 2 with a red scale bar of 500 μm; **(F)** The BAM of DOI 2; **(G)** The BVS of DOI 2; **(H)** The TI skeleton map of DOI 2.

This automatic algorithm firstly would detect the end depth, where all OCTA signals attenuated and only noise remained, by finding the deepest location with the same intensity of the mean intensity of all depths. The layer separation depth was considered to be the location of the maximum intensity peak. Therefore, DOI 1 was selected from the top to the layer separation depth, while DOI 2 was selected from the layer separation depth to the end depth. With two layers separated as DOI 1 and 2, both can be used to generate the intermediate maps for quantitative assessments which is shown in Figure 5. The OCTA quantitative metrics would be calculated from the intermediate maps for both DOIs.

As shown in Figure 2, the binary images were generated from the image processing, and then were used to calculate the OCTA quantitative metrics. For VAD, the area of the blood vessels divided by the whole area of the field of view was utilized as the density of the vascular area, which can be shown as Equation 1,

$$\text{VAD}=\frac{\sum_{x=1}^{X}\sum_{y=1}^{Y}\text{BA}M_{\left(x,y\right)}}{X\times Y}\times 100\%$$ (1)

where X and Y are the number of pixels on two axes of the *en face* projection images, x and y are the coordinates of the binary mask (Reif et al., 2012; Jia et al., 2015). VSD was the density of the vessel skeleton areas, which can be calculated as Equation 2.

$$\text{VSD}=\frac{\sum_{x=1}^{X}\sum_{y=1}^{Y}\text{BV}S_{\left(x,y\right)}}{X\times Y}\times 100\%$$ (2)

As metrics of density, VAD and VSD were both presented in percentages (Reif et al., 2012; Agemy et al., 2015; Chu et al., 2016). Both the BAM and BVS were used to calculate the VDI. The ratio of the blood vessels‘ areas to the vessel skeletons was defined as VDI which is shown in Equation 3,

$$\text{VDI}=\frac{\sum_{x=1}^{X}\sum_{y=1}^{Y}\left(\text{BV}S_{\left(x,y\right)}\times dis_{Eu_{\left(x,y\right)}}\times 2\right)}{\sum_{x=1}^{X}\sum_{y=1}^{Y}\left(\text{BV}S_{\left(x,y\right)}\right)}$$ (3)

where $dis_{Eu_{\left(x,y\right)}}$ is the shortest Euclidean distance from the pixel (x, y) to the vessel edge for all pixels on the vessel skeletons (Maurer et al., 2003). The unit of VDI is pixels, while it can further be converted into micrometers using the pixel transverse size, which was done for all results in this study (Chu et al., 2016). TI represents the tortuosity of the blood vessels, which can be calculated by the average ratio of the vessel length divided by the Euclidean distance for all vessel segments (Lee et al., 2018; Martelli and Giacomozzi, 2021), which is shown in Equation 4,

$$\text{TI}=\left(\frac{SegmentLength}{EuclideanDistance}-1\right)\times 100$$ (4)

where SegmentLength and EuclideanDistance are respectively the length and the Euclidean distance of each vessel segment. After calculating the TI values for all vessel segments, a TI skeleton map can be generated. However, in the analysis of vascular tortuosity, it is essential to account for the varying physiological significance of blood vessels of different diameters (Han, 2012; Yoon et al., 2023). This approach aligns with established practices in vascular research where the relative contribution of each vessel is proportionate to its diameter, reflecting its functional importance (Han, 2012; Yoon et al., 2023). Therefore, we utilized a weighted TI (WTI) method, that uses the diameter of each vessel segment as a weight parameter during the averaging which is shown in Equation 5,

$$\text{WTI}=\frac{\sum_{n=1}^{N}VDI_{n}\times \left(\frac{SegmentLength_{n}}{EuclideanDistance_{n}}-1\right)}{\sum_{n=1}^{N}VDI_{n}}\times 100$$ (5)

where $VDI_{n}$ is the VDI of the n th separated vessel segment, N is the total number of the vessel segments, $SegmentLength_{n}$ and $EuclideanDistance_{n}$ are respectively the length of the n th vessel segment and the Euclidean distance of the two endpoints of the n th vessel segment. WTI does not have a unit. To the best of our knowledge, this is the first instance where WTI has been employed to quantify the morphological characteristics of the oral microvasculature using OCT, although other metrics, such as vessel complex index, fractal dimension, and VI have been previously used in ophthalmological applications (Reif et al., 2012; Chu et al., 2016; Lee et al., 2018; Martelli and Giacomozzi, 2021). The proposed metric, WTI, to assess the tortuosity of blood vessels was evaluated, which was introduced in the Appendix.

## 3 Results

### 3.1 Quantitative maps in a healthy case

**FIGURE 6:**
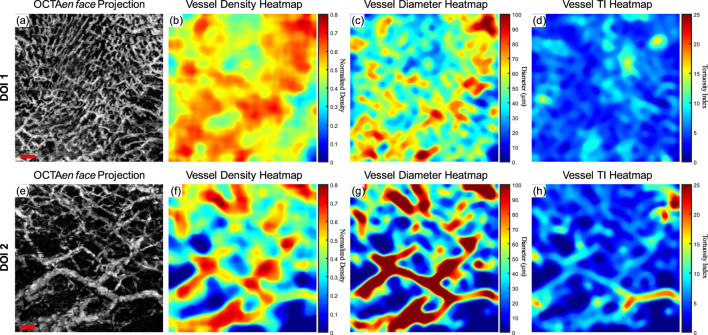
The quantitative maps of a normal labial mucosa dataset. **(A)** The gray-scale OCTA *en face* projection of the DOI 1 segmented by the automatic DOI selection algorithm with a scale bar of 500 μm; **(B)** The quantitative heatmap of vessel density of **(A)**, which was calculated using a moving kernel; **(C)** The quantitative heatmap of vessel diameter in micrometers of **(A)** using a moving kernel; **(D)** The quantitative heatmap of TI of **(A)** using a moving kernel; **(E)** The gray-scale OCTA *en face* projection of the DOI 2 segmented by the automatic DOI selection algorithm with a scale bar of 500 μm; **(F)** The quantitative heatmap of vessel density of **(E)**, which was calculated using a moving kernel; **(G)** The quantitative heatmap of vessel diameter in micrometers of **(E)** using a moving kernel; **(H)** The quantitative heatmap of TI of **(E)** using a moving kernel.

A clinically healthy labial mucosa dataset was shown in Figure 6 as a demonstration of the quantitative assessments of oral tissue OCTA imaging. Figure 6A shows the gray-scale OCTA *en face* projection of DOI 1, which was segmented by the automatic DOI selection algorithm. Figure 6B illustrates the quantitative heatmap of vessel density of Figure 6A, which was generated by using a moving kernel calculating the average vessel density within the kernel. All quantitative heatmaps were generated using the same method. Figure 6C displays the quantitative heatmap of vessel diameter in micrometers of Figure 6A, while Figure 6D shows the quantitative heatmap of TI of Figure 6A. Similarly, Figures 6E–H presents the same quantitative results of DOI 2.

Using the quantitative assessment methods above, VAD, VSD, VDI, and WTI were calculated from the OCTA projection images. For DOI 1, this healthy labial mucosa dataset output VAD of 53.26%, VSD of 7.00%, VDI of 91.04 μm, and WTI of 20.30. Within DOI 2, the VAD and VSD decreased to 46% and 5.40%, which corresponded to the visual observation of the grayscale OCTA projection. An increase in the vessel diameter in DOI 2 also saw the VDI’s rise, while the WTI decreased to 18.36 in DOI 2.

### 3.2 Evaluation of repeatability

**FIGURE 7:**
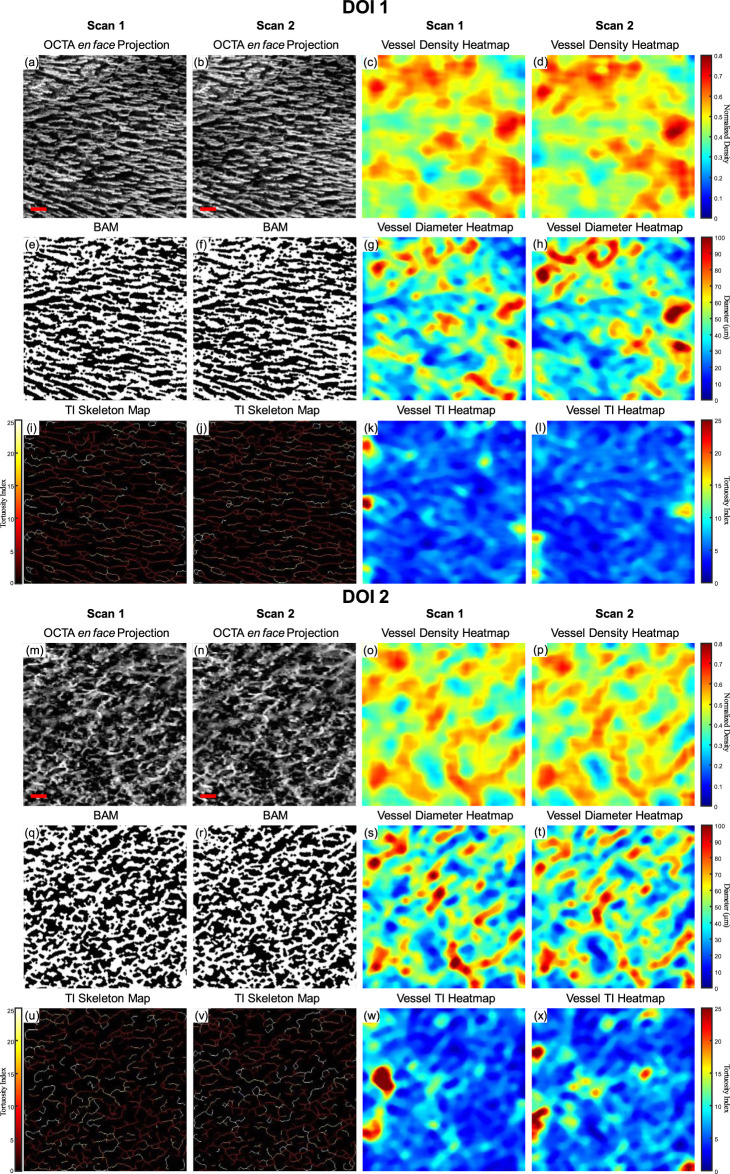
A repeatability test for two repeated scans with buccal mucosa. **(A)** The greyscale OCTA *en face* projection of DOI 1 in Scan 1; **(B)** The greyscale OCTA *en face* projection of DOI 1 in Scan 2; **(C)** The quantitative heatmap of vessel density of **(A)**; **(D)** The quantitative heatmap of vessel density of **(B)**; **(E)** The BAM of **(A)**; **(F)** the BAM of **(B)**; **(G)** The quantitative heatmap of vessel diameter of **(A)**; **(H)** The quantitative heatmap of vessel diameter of **(B)**; **(I)** The TI skeleton map of **(A)**; **(J)** The TI skeleton map of **(B)**; **(K)** The quantitative heatmap of TI of **(A)**; **(L)** The quantitative heatmap of TI **(B)**; **(M)** The greyscale OCTA *en face* projection of DOI 2 in Scan 1; **(N)** The greyscale OCTA *en face* projection of DOI 2 in Scan 2; **(O)** The quantitative heatmap of vessel density of **(M)**; **(P)** The quantitative heatmap of vessel density of **(N)**; **(Q)** The BAM of **(M)**; **(R)** The BAM of **(N)**; **(S)** The quantitative heatmap of vessel diameter of **(M)**; **(T)** The quantitative heatmap of vessel diameter of **(N)**; **(U)** The TI skeleton map of (m); **(V)** The TI skeleton map of **(N)**; **(W)** The quantitative heatmap of TI of **(M)**; **(X)** The quantitative heatmap of TI of **(N)**. All scale bars represent 500 μm.

As a demonstration for the repeatability of the quantitative assessment of OCTA imaging on oral tissues, a side-by-side comparison of two successive OCTA acquisitions was shown in Figure 7. The greyscale OCTA *en face* projection of DOI 1 is shown in Figures 7A, B, corresponding to Scan 1 and Scan 2 respectively. The corresponding quantitative heatmaps of vessel density are illustrated in Figures 7C, D. The BAMs of these scans are depicted in Figures 7E, F. Additionally, the quantitative heatmaps of vessel diameter for Scan 1 and Scan 2 are presented in Figures 7G, H, respectively. The TI skeleton maps of Scan 1 and Scan 2 are shown in Figures 7I, J, with the quantitative heatmaps of TI for these scans illustrated in Figures 7K, L. Similarly, the greyscale OCTA *en face* projections and quantitative results of DOI 2 are displayed in Figures 7M–X.

The quantitative maps shared the visual similarity between two successive scans. The quantitative metrics were calculated for both Scan 1 and Scan 2 as well, which were listed in Table 1. In addition, the coefficient of variation for each metric was calculated between two scans, which was considered to have reliable repeatability according to published standard (Chu et al., 2016).

**TABLE 1:** Quantitative analysis of two repeated scans with buccal mucosa.

Depth of interest	DOI 1				DOI 2			
Quantitative Metrics	VAD	VSD	VDI [μm]	WTI	VAD	VSD	VDI [μm]	WTI
**Scan 1**	49.58%	7.12%	85.65	12.86	47.58%	6.27%	91.89	21.34
**Scan 2**	50.05%	7.38%	84.95	12.40	47.59%	6.26%	91.48	20.77
**Coefficient of variation**	0.0047	0.0179	0.0041	0.0257	0.0001	0.0008	0.0022	0.0194

### 3.3 Building database for healthy subjects

Aggregating a database of normal oral tissue OCTA ran concurrently within this study. A large database can be used for quantitative assessment to establish a guideline of the quantitative metrics for healthy oral tissue, which can be used to compare diseased cases in future studies. Currently, 32 healthy participants have been enrolled for buccal mucosa acquisitions. The quantitative metrics of buccal mucosa datasets were listed in Table 2. In addition, 24 healthy individuals have participated in the collection of labial mucosa datasets, and the corresponding quantitative metrics are provided in Table 3. Furthermore, 13 healthy subjects have been included in the acquisition of datasets from the floor of the mouth, with the metrics outlined in Table 4. Lastly, eight healthy participants have been involved in the acquisition of hard palate datasets, with the quantitative metrics presented in Table 5.

**TABLE 2:** The quantitative metrics of buccal mucosa datasets. (n = 32).

Depth of interest	DOI 1				DOI 2			
Quantitative Metrics	VAD	VSD	VDI [μm]	WTI	VAD	VSD	VDI [μm]	WTI
**Mean**	43.32%	6.55%	74.50	20.45	41.17%	5.64%	83.09	22.17
**Standard Deviation**	8.12%	1.33%	10.79	3.16	6.73%	1.04%	9.64	2.23
**Lower 95%**	40.39%	6.08%	70.61	19.31	38.75%	5.27%	79.61	21.37
**Upper 95%**	46.25%	7.03%	78.38	21.59	43.60%	6.02%	86.57	22.97

**TABLE 3:** The quantitative metrics of labial mucosa datasets. (n = 24).

Depth of interest	DOI 1				DOI 2			
Quantitative Metrics	VAD	VSD	VDI [μm]	WTI	VAD	VSD	VDI [μm]	WTI
**Mean**	49.97%	6.64%	87.76	18.86	44.11%	4.89%	108.41	16.82
**Standard Deviation**	5.18%	0.86%	9.20	3.16	5.46%	0.79%	13.48	2.62
**Lower 95%**	47.78%	6.28%	83.88	17.53	41.81%	4.55%	102.71	15.71
**Upper 95%**	52.16%	7.00%	91.65	20.20	46.42%	5.23%	114.10	17.92

**TABLE 4:** The quantitative metrics of the floor of the mouth datasets. (n = 13).

Depth of interest	DOI 1				DOI 2			
Quantitative Metrics	VAD	VSD	VDI [μm]	WTI	VAD	VSD	VDI [μm]	WTI
**Mean**	52.00%	5.68%	120.21	17.18	53.98%	4.64%	161.67	15.41
**Standard Deviation**	5.28%	1.09%	23.07	2.05	5.93%	0.61%	31.62	2.07
**Lower 95%**	48.81%	5.02%	106.27	15.94	50.40%	4.27%	142.57	14.09
**Upper 95%**	55.19%	6.33%	134.15	18.41	57.57%	5.01%	180.78	16.73

**TABLE 5:** The quantitative metrics of the hard palate datasets. (n = 8).

Depth of interest	DOI 1				DOI 2			
Quantitative Metrics	VAD	VSD	VDI [μm]	WTI	VAD	VSD	VDI [μm]	WTI
**Mean**	33.19%	4.33%	85.25	23.39	35.83%	3.94%	114.90	19.69
**Standard Deviation**	6.07%	1.25%	8.88	4.89	4.92%	0.96%	26.53	3.87
**Lower 95%**	28.12%	3.29%	77.82	19.30	31.71%	3.13%	92.72	16.45
**Upper 95%**	38.27%	5.37%	92.67	27.48	39.95%	4.74%	137.07	22.93

### 3.4 Quantitative analysis of microvascular in benign labial ulcer

**FIGURE 8:**
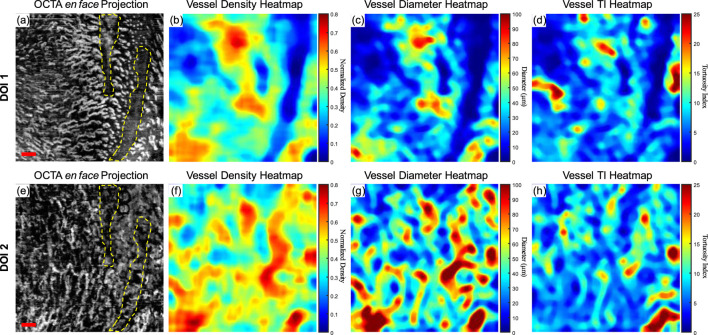
The quantitative maps of a labial mucosa dataset with a benign ulcer. **(A)** The gray-scale OCTA *en face* projection of the DOI 1 segmented by the automatic DOI selection algorithm with a scale bar of 500 μm; **(B)** The quantitative heatmap of vessel density of **(A)**, which was calculated using a moving kernel; **(C)** The quantitative heatmap of vessel diameter in micrometers of **(A)** using a moving kernel; **(D)** The quantitative heatmap of TI of **(A)** using a moving kernel; **(E)** The gray-scale OCTA *en face* projection of the DOI 2 segmented by the automatic DOI selection algorithm with a scale bar of 500 μm; **(F)** The quantitative heatmap of vessel density of **(E)**, which was calculated using a moving kernel; **(G)** The quantitative heatmap of vessel diameter in micrometers of **(E)** using a moving kernel; **(H)** The quantitative heatmap of TI of **(E)** using a moving kernel.

Although a large database of oral mucosal tissue in diseased states would be needed in future, an abnormal dataset was presented below in comparison with the quantitative results of normal database. Figure 8 shows the quantitative heatmaps of a labial mucosa dataset from a participant who developed a benign oral ulcer at the time of acquiring the dataset in Figure 8. The ulcer area was highlighted with the dashed lines. The 3D OCTA dataset was separated into two DOIs, and generated heatmaps of vessel density, vessel diameter, and vessel TI, in the same way of Figure 6.

Compared to the healthy labial mucosa dataset results, the ulcer metrics reveal distinct differences across various parameters. For DOI 1, the ulcer’s VAD of 35.07% is lower than the healthy mean VAD of 49.97%, indicating a reduced density of blood vessels in the ulcerated tissue which can also be visually observed in the grayscale OCTA projection. For DOI 2, the ulcer’s VAD of 46.24% is closer to the healthy mean of 44.11%, but still slightly elevated, suggesting a potential increase in vascular proliferation in the ulcerated region at greater depths. The VSD for DOI 1 in the ulcer case is 5.10%, which is lower than the healthy mean of 6.64%, reflecting fewer vessel segments in the ulcerated tissue which corresponds to the OCTA projection. For DOI 2, the ulcer’s VSD of 5.60% is higher than the healthy mean of 4.89%, suggesting an increased segmentation in the vessel network at this depth. The ulcer’s VDI for DOI 1 is 77.37 μm, lower than the healthy mean of 87.76 μm, indicating narrower vessel diameters in the ulcerated area. Similarly, for DOI 2, the ulcer’s VDI of 101.71 μm is lower than the healthy mean of 108.41 μm. The WTI for DOI 1 in the ulcer dataset is 22.13, higher than the healthy mean of 18.86, suggesting increased vessel tortuosity in the ulcerated tissue. For DOI 2, the ulcer’s WTI of 25.02 is higher than the healthy mean of 16.82, indicating even greater tortuosity in the deeper vessels of the ulcerated region.

These detailed comparisons reveal that the ulcerated labial mucosa exhibits alterations in microvascular metrics compared to healthy tissue, with notable differences in vessel density, diameter, and tortuosity, reflecting the pathological changes associated with ulceration. However, to reach a statistical conclusion, more datasets of ulceration would be required to perform a statistical analysis. This ulceration case only demonstrates the diagnostic potential of quantitative assessment methods for oral microvasculature in this study.

## 4 Discussion

In this study, we demonstrated the use of OCTA to image and quantify microvasculature in *in vivo* human healthy and abnormal oral cavity. Four metrics, including vessel area density (VAD), vessel skeleton density (VSD), vessel diameter index (VDI), and the newly proposed weighted tortuosity index (WTI) were employed for the quantitative assessment of oral OCT angiograms. The microvasculature in the superficial layer (DOI 1) and deeper layer (DOI 2) of four intraoral sites, involving buccal mucosa, labial mucosa, floor of the mouth and hard palate, were quantified and contributed to the construction of a database from healthy participants. Additionally, a microvasculature analysis of a benign labial ulcer indicated that the metrics differed from those in the healthy dataset.

This study has several achievements. Firstly, we developed a robust method to automatically segment the superficial and deep layers of the oral cavity OCTA volume. Secondly, we introduced the WTI metric for accurate tortuosity quantification. Thirdly, we showcased the repeatability of our quantitative metrics results using a normal buccal mucosa case scanned successively at the same location. Lastly, and importantly, to the best of our knowledge, this is the first study to provide quantitative metrics for *in vivo* healthy and abnormal oral OCT angiograms. This work has the potential to offer clinicians a rapid and comprehensive strategy for interpreting angiograms.

Microvasculature has been shown to differ significantly between tissue layers, with clear boundaries typically segmented layers in dermatology studies (Men et al., 2017; Meiburger et al., 2019) and retinal studies (Garrity et al., 2017; Zhou et al., 2020). However, in the oral cavity, particularly within mucosal tissues, the epithelium and lamina propria are not well differentiated under OCT imaging (Feldchtein et al., 1998), posing challenges for segmentation and subsequent blood vessel overlapping, which causes inaccurate quantification. In addition, the epithelial thickness in oral soft and hard tissues can vary largely, even within the same site (Di Stasio et al., 2019). Manual segmentation of these tissues is often impractical for large datasets due to its labor-intensive nature (Hill et al., 2024). In our study, we proposed a depth segmentation method based on the maximum OCTA intensity value derived from the overall mean intensity across all depths. With the proposed method, the overlapping issue in Figure 3 was reduced, which was shown in Figure 5. Although the proposed method was based on the OCTA intensity, not biological features, we are developing a deep-learning tool capable of automatically segmenting the epithelial layer for future work, which will enhance efficiency and accuracy.

Quantitative metrics play an important role in understanding OCT angiograms. Many studies employed only a single index to analyze the angiogram, such as VAD (Men et al., 2017), vessel diameter index (VDI) (Yao et al., 2021), or tortuosity index (TI) (Martelli and Giacomozzi, 2021). In contrast, our study utilized multiple metrics, providing a more comprehensive understanding of vasculature from various perspectives. Additionally, we proposed and evaluated a new metric, the vessel tortuosity index (WTI), which considered the factor of vessel diameter. To understand the quantitative metrics for a healthy oral database, we found that the VAD in the buccal mucosa (DOI1: 43.32%; DOI 2:41.17%), labial mucosa (DOI 1: 49.97%; DOI 2: 44.11%), and floor of the mouth (DOI 1: 52.00%; DOI 2: 53.98%) were similar, while the VSD of the buccal mucosa (DOI 1: 6.55%; DOI: 5.64%), labial mucosa (DOI 1: 6.64%; DOI 2: 4.89%), and floor of the mouth (DOI 1: 5.68%; DOI 2: 4.64%) were found in close values, suggesting these areas had comparable blood vessel density and distribution. This similarity was likely due to their roles as oral mucosa with similar functions in protecting the underlying tissue and facilitating oral movements (Berkovitz, 2009; Nanci, 2012). The hard palate exhibited the smallest VAD (DOI 1: 33.19%; DOI 2: 35.83%) and VSD (DOI 1: 4.33%; DOI 2: 3.94%) in four oral sites, along with a higher vessel diameter (DOI 1: 85.25 μm; DOI 2: 114.90 μm) and lower tortuosity (DOI 1: 23.39; DOI 2: 19.69) in its deep layer compared to the superficial layer. These characteristics are possibly related to its specific anatomical features and the presence of numerous minor salivary glands between the mucosal surface and the underlying bone (Berkovitz, 2009; Nanci, 2012). The floor of the mouth had the largest VDI (DOI 1: 120.21 μm; DOI 2: 161.67 μm) but the smallest WTI (DOI 1: 17.18; DOI 2: 15.41), which may be attributed to its larger vasculature and unique structural characteristics. In comparison, the ulcer dataset showed a decrease in superficial capillary VAD (DOI 1: 35.07%) and VDI (DOI 1: 77.37 μm), indicating structural damage (Xie et al., 2024). The WTI in the ulcer (DOI 1: 22.13; DOI 2: 25.02) was higher than in healthy labial mucosa (DOI 1: 18.86; DOI 2: 16.82), suggesting increased vessel tortuosity in the ulcerated tissue. This increased tortuosity may indicate a response to inflammation and tissue repair mechanisms (Chong et al., 2017). A common early clinical state of OSCC is an ulcerated lesion (Pires et al., 2013). Therefore, our quantification framework could offer valuable insights into microvascular differences among multiple oral sites and changes during the progression of oral disease.

In addition to comprehensively identifying quantitative differences from the OCTA images among the healthy database and pathological condition of the oral cavity, the repeatability test of metrics exhibited high consistency of our methods. The low coefficient of variation (CV) values indicate that our quantification method was relatively repeatable between scans (CV < 5%), and the common indexes were smaller than previously published quantification works (Chu et al., 2016; Xie et al., 2024). Therefore, our methods should provide a high level of accuracy when using OCTA for monitoring oral disease progression and treatment response.

A few limitations of this study need to be addressed. First, since this study was using *in vivo* OCTA imaging, there is a lack of histological validation. Histological analysis, such as using Griffonia simplicifolia lectin (GSL) to visualize vascular lumens (Xie et al., 2024), could be conducted on biopsies from diseased patients. Additionally, to achieve more accurate descriptive statistics for healthy subjects, a larger sample size will be necessary. This limitation aligns with our intended future work, which aims to better understand normal tissue compared with biopsy proven clinically abnormal tissues. Larger sample sizes of both healthy and diseased tissue will enable us to conduct more robust statistical analyses and more accurately determine the utility of OCTA in oral diagnostics using our methods. A minimum of sample sizes of patients and healthy volunteers of 90 and 180 respectively would be ideal to yield 80% power with a significance level of 0.05. While our proposed depth segmentation method was reliable for OCTA quantification, the segmentation of actual layers in the oral cavity can reveal structural differences of multiple oral sites and structural changes caused by oral diseases. Considering the challenges of using conventional image processing to differentiate layers, deep-learning methods have the potential to accurately segment the oral cavity (Hill et al., 2024).

## 5 Conclusion

In this study, we demonstrated a comprehensive method to quantify the microvasculature in the oral cavity, including buccal mucosa, labial mucosa, floor of the mouth and hard palate. From the intraoral scanning results, we assessed four metrics: VAD, VSD, VDI, and the newly proposed WTI. These metrics were calculated in both superficial and deeper layers across four intraoral sites, using an automatic layer separation method. We also analyzed a benign ulcerated labial tissue. The four metrics collectively revealed differences from healthy tissue. Therefore, proposed quantitative assessment of OCTA imaging holds considerable promise for future research and clinical management of oral diseases.

## Figures and Tables

**FIGURE A1:**
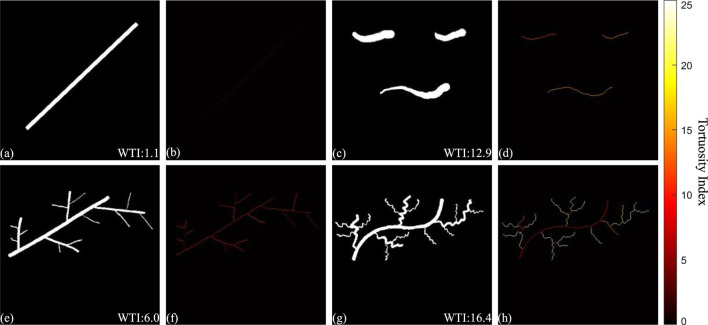
Demonstration of the quantitative assessment of vasculature tortuosity. Figure **(A, C, E, G)** are hand-drawn binary images with vessel-like shapes, with WTI values calculated and displayed at the bottom-right corner. Figure **(B, D, F, H)** are the TI skeleton maps of the binary images respectively.

## Data Availability

The datasets presented in this article are not readily available because of ethical restrictions. Requests to access the datasets should be directed to Chunhui Li, c.li@dundee.ac.uk.

## Appendix

To evaluate the robustness of the WTI and better demonstrate WTI, four hand-drawn binary images which essentially can simulate the simplified binarized *en face* OCTA projections, BAM, were used to calculate the WTI and generate TI skeleton maps (Figure A1). Figures A1A, C, E, H are the hand-drawn binary images with vessel-like shapes to evaluate the tortuosity quantification. The WTI values were calculated and displayed at the bottom-right corner on each sub-figure. The first two images, Figures A1A, C were designed to assess separated segments, where one was a straight line and another consisted of curved lines. Figures A1B, D were the TI skeleton maps of Figures A1A, C respectively. Besides the separated segments, connected vessel-like binary images in Figures A1E, G were applied to the same process. Figure A1E was a connected vessel branch with relatively straight lines, while Figure A1G was the same structured vessel branch with visually more tortuous vessels. Figures A1F, H were the TI skeleton maps of the binary images in Figures A1E, G. In comparisons of either separated segments or connected vessel branches, a positive relationship between the visual tortuosity and the WTI values was found in this demonstration. Therefore, the WTI can be considered as a useful tool to quantitatively assess the tortuosity of the microvasculature networks.
